# Water walking as a new mode of free surface skipping

**DOI:** 10.1038/s41598-019-42453-x

**Published:** 2019-04-15

**Authors:** Randy C. Hurd, Jesse Belden, Allan F. Bower, Sean Holekamp, Michael A. Jandron, Tadd T. Truscott

**Affiliations:** 1 Utah State University, Department of Mechanical & Aerospace Engineering, Logan, UT 84321 USA; 2 Naval Undersea Warfare Center, Newport, RI 02841 USA; 3 Brown University, School of Engineering, Providence, RI 02912 USA

## Abstract

Deformable elastomeric spheres are evaluated experimentally as they skip multiple times over a lake surface. Some spheres are embedded with small inertial measurement units to measure the acceleration experienced during water surface impact. A model for multiple impact events shows good agreement between measured acceleration, number of skipping events and distanced traveled. The experiment reveals a new mode of skipping, “water walking”, which is observed for relatively soft spheres impacting at low impact angles. The mode occurs when the sphere gains significant angular velocity over the first several impacts, causing the sphere to maintain a deformed, oblong shape. The behavior is characterized by the sphere moving nearly parallel to the water surface with the major axis tips dipping below the water surface with each rotation while the shorter sides pass just above, giving the impression that the sphere is walking across the water surface.

## Introduction

As early as the late 16th Century, naval gunners knew that a cannonball fired at a sufficiently small impact angle to the water surface could be made to skip across the sea^1^. The technique simplified targeting because skipping spheres would strike any object along a straight line, rather than only a single point. By the mid 19th century, engineers were investigating how changes in impact angle and sphere density affected the number of skipping events. Notes from experiments collected in 1855 record a 24.5 kg sphere skipping 32 times before entry^2^. In 1883, de Jonquières discussed that a critical impact angle exists above which skipping does not occur, which is inversely proportional to the square root of the sphere specific gravity (${\theta }_{c}=18/\sqrt{SG}$)^3^.

Early efforts to study skipping behavior produced little more than qualitative results^4^. In 1944, photographic techniques allowed Birkhoff *et al*. to perform a more complete study and create a model for the forces experienced by a rigid sphere obliquely impacting a liquid surface^5^. However, Birkhoff’s model is not completely analytical. Other researchers, as well as Birkhoff himself, have noted that more than one assumption in the model is not necessarily true, but provides a good estimate of the forces involved^4^. Johnson mentions that, “the phenomenon of ricochet [on water] is well known but not widely understood because it takes place in circumstances difficult to control and define… It is a succession of oblique high speed impact events… in which the geometry of each individual strike is different”. Perhaps for this reason, most studies concerning skipping phenomenon focus on single impacts rather than the complete skipping event^4^. Hewitt *et al*. studied subsequent water skipping events, more specifically a fixed plane skipping on a moving channel^6^. The authors identified multiple skipping states or modes and presented several models to explain their qualitative features. While the governing principles are similar, the study by Hewitt *et al*. differs significantly from an investigation of a freely flying body on a fixed liquid pool. More recently, skipping behavior has been studied in reference to skipping stones. In 2003, Bocquet presented a model which he used to theoretically estimate the maximum number of skips that can be achieved with a skipping stone^7^. Clanet *et al*. published an experimental investigation in 2004 which identified optimal values for both impact angle and attack angle^8^. The model for stone skipping was refined further in light of experimental results by Rosellini *et al*.^9^. In 2005, Nagahiro and Hayakawa performed a three-dimensional simulation of stone skipping which produced results in agreement with those discovered by Clanet *et al*.^10^.

Deformable skipping spheres were first studied in 2012 when Truscott *et al*. investigated a water skipping toy known as a Waboba^®^^11^. They noted that deformation upon impact appears to increase lift and thus produce more successful skipping. Following publications by the same research group have provided additional insight into why elastomeric spheres skip more readily than their rigid counterparts^12,13^. Deformation upon impact not only increases cross-sectional area, resulting in greater lift, but also produces more favorable attack angles with the water surface. These previous studies focus primarily on isolated single skip events and only present data collected in a laboratory setting. They did study short multi-skip events (less than four skips), with each impact inducing a predictable deformation mode and type of skip. However, they do not address long multiple skipping events (i.e., number of skips $\gg 4$) where one may want to understand how the dynamics change in later skips, or to predict number of skips and distance traveled. As will be shown herein, the physics can indeed be different and require alterations to existing models to describe the observed phenomena.

This paper presents an investigation of the skipping behavior of elastomeric spheres over successive skipping events. The measured results are compared to predictions from a model validated for single skips and extended here to multiple skipping events. Additionally, experimental investigations revealed a previously unknown mode of water surface skipping for elastomeric spheres. The new skipping mode consists of the sphere moving nearly parallel to the water surface while impacting the water rapidly; this gives the impression that the sphere is walking across the water surface. We investigate the causes and behavior of this newly observed phenomenon.

## Results

### Multiple skip phenomena

**Figure 1:**
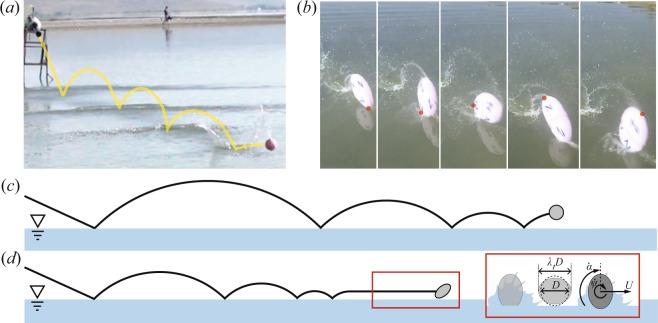
(**a**) An elastomeric sphere skips from the water surface several times in a traditional manner with the distance between skips decreasing significantly with each impact event; a yellow line traces the sphere trajectory. (**b**) An elastomeric sphere exhibits the “water walking” skipping mode. A red dot identifies the same point throughout the image sequence (4 ms between images). The sphere rotates at high speeds, maintaining a deformed shape, with only the tips of the major axis of the deformed spheroid dipping below the surface. (**c**) A side view of a characteristic trajectory for a traditional skipping event. (**d**) A side view of a characteristic trajectory for a water walking skipping event. Sphere deformation is indicated by the parameter *λ*_1_, which is experimentally measured from the major axis. Rigid rotation rate is represented by the term $\dot{\psi }$.

A deformable elastomeric sphere impacting a water surface at an oblique angle will, under certain conditions, rebound or skip from the water surface. When this event happens in a large space, the rebounding sphere follows a predictable projectile trajectory before impacting the water a second time^13^. Depending on the initial impact conditions the sphere can skip once, twice, or several times before losing the energy necessary to skip and (for spheres denser than water such as these) entering the water. The height and distance achieved between skips decreases with each skipping event, just like a skipping stone, with the last few skips before entry typically occurring rapidly with spacing between impact events near one sphere diameter. These rapid impacts just prior to entry are known as “pitty-pat” in the stone skipping community. A photographic example of this type of traditional skipping for elastomeric spheres is presented in Fig. 1(a). A representative trajectory of traditional skipping is presented in Fig. 1(c).

In addition to the skipping behavior described above, the skipping experiments performed at the lake showed that when softer spheres (smaller *G*) were skipped with lower values of impact angle *β*_0_, they would often transition from traditional skipping behavior (always within 3–4 skips), to moving approximately parallel to the water surface with impact events occurring very close together (on the order of one sphere diameter). Photographs of this unique skipping behavior are shown in Fig. 1(b), and it is diagramed in Fig. 1(d). In this mode, the height of skipping events was nearly indiscernible, even from high speed footage. This motion parallel to the water surface, with rapid impact events, gave the impression that the sphere was “walking” across the water surface in a manner reminiscent of the water walking basilisk lizard^14^. Upon closer inspection of high speed photographs, we observed that spheres demonstrating this behavior exhibited high angular velocity $\dot{\psi }$ and generally maintained a more pronounced deformed shape during the skipping event (visible in Fig. 1(b)). This deformation is represented by the parameter *λ*, shown in the inset of Fig. 1(d).

**Figure 2:**
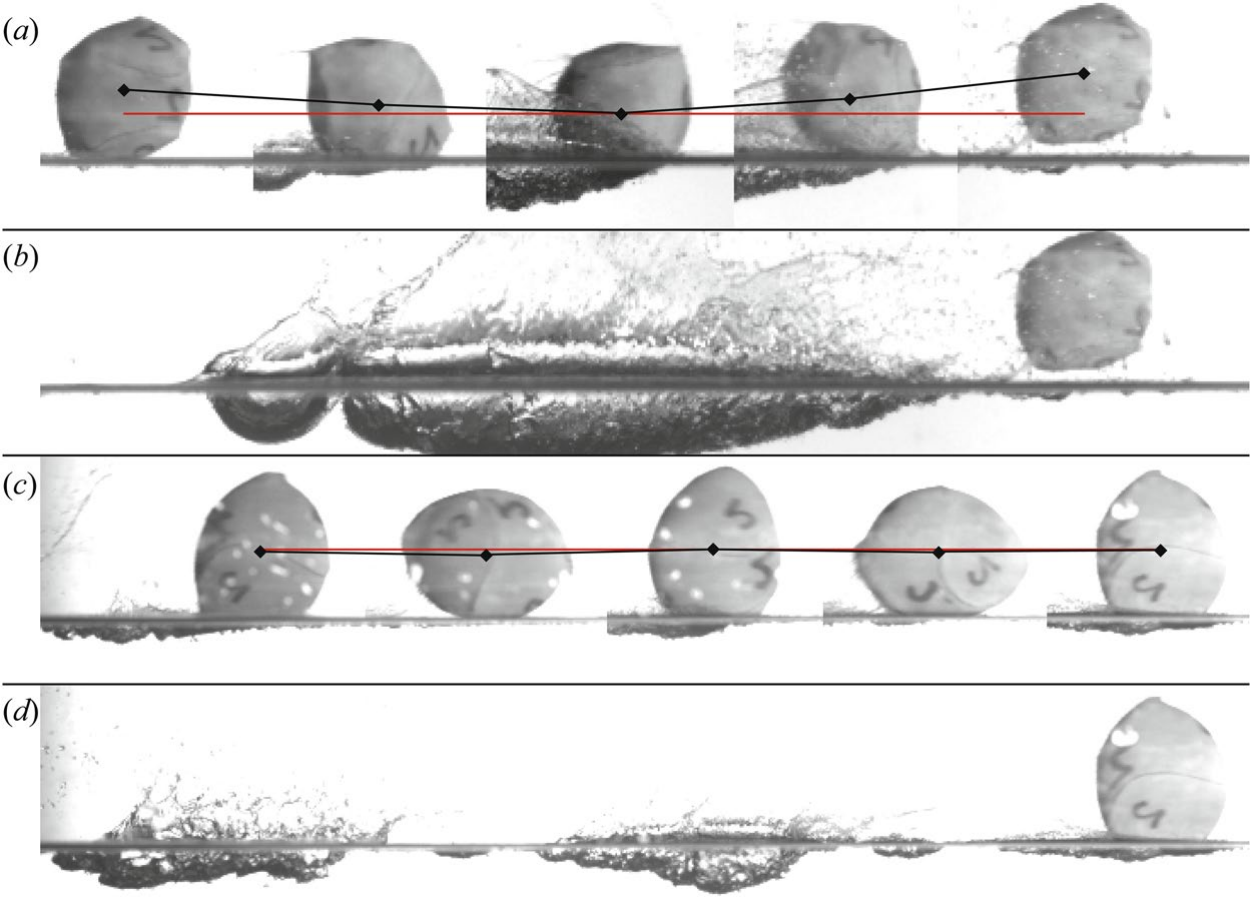
High speed images of two types of skipping events are shown. The skipping events were filmed in a long glass tank using high speed cameras and diffuse backlighting. (**a**) A traditional skipping event in which the sphere impacts the free surface at an oblique angle and creates a single cavity before rising above the free surface (12 ms between images). The sphere completes one full rigid rotation in the period *T*, which begins at the first displayed black diamond and ends at the last. The black diamonds mark the sphere centroid through the image sequence and red lines are parallel to the water surface. Measurements of angular velocity $\dot{\psi }$ are averaged over one period *T*. For the event shown in (**a**), *T* = 48 ms and $\dot{\psi }=130.9$ rad/s. (**b**) The cavity formed by the sequence shown in (**a**) is relatively long and deep. (**c**) A different type of sphere skipping is shown where the sphere centroid moves nearly parallel to the water surface and only the tips of the major axis of the prolate spheroid create cavities (6 ms between images, *T* = 24 ms, $\dot{\psi }=261.8$ rad/s). (**d**) Only the tips of the major axis of the prolate sphere strike the water surface resulting in two distinct, shallow cavities formed during one rotation period. This type of skipping is referred to as “water walking”.

We investigate the differences between this “water walking” behavior and traditional skipping through high speed photographs collected in a more controlled lab setting. Consider the traditional skipping event in shown Fig. 2(a); the ball impacts at a shallow oblique angle, creates a single cavity, then rises above the water surface. The centroid of the sphere image (black diamond) is clearly descending before impact and ascending after impact. The cavity created by this impact sequence is shown in Fig. 2(b). By contrast, the sphere in Fig. 2(c) exhibits water walking. The sphere maintains a more pronounced deformed shape and impact events appear not to come from the sphere descending into the water, but due to the tips of the major axis of the deformed spheroid rotating to interact with the water surface (*d*). This impact event doesn’t generate enough lift to cause the ball to ascend significantly, but generates sufficient lift to prevent the sphere from descending below the water surface (note the relatively constant height of the centroid). In an extreme case, this behavior was observed to persist for more than 100 surface impact events.

### Modeling multiple skip events

**Figure 3:**
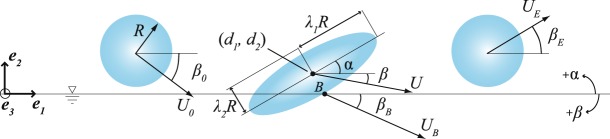
An elastomeric sphere is represented before, during and following impact with a water surface. The sphere radius is represented by *R* where *λ*_1_ and *λ*_2_ represent the principal material stretches. The magnitude and direction of sphere velocity are represented by *U* and *β*, with subscripts referring to those values prior to impact (0), at the sphere bottom during impact (*B*) and exiting the water (*E*). The location of the sphere centroid is described by (*d*_1_, *d*_2_) and *α* is the attack angle during impact.

This newly observed skipping behavior, as well as traditional skipping events, are investigated in light of the hydrodynamic force model proposed by Belden *et al.*^13^. Consider an elastomeric sphere impacting the water surface as shown in Fig. 3. The magnitude and direction of sphere velocity are represented by *U* and *β*, with subscripts referring to those values prior to impact (0), at the sphere bottom during impact (*B*) and upon exiting the water (*E*). Sphere deformation during impact is represented by principal stretches *λ*_1_ and *λ*_2_, which describe the ratio of the deformed spheroid axes to the undeformed sphere radius *R* (see Figs 1(d) and 3). The coordinates of the sphere centroid are (*d*_1_, *d*_2_) and the attack angle is represented by *α* and is set by the orientation of the principal stretches of the deformed sphere. The sphere is idealized as an incompressible neo-Hookean hyperelastic solid with shear modulus *G* and density ${\rho }_{s}$. Belden *et al*. altered an existing model proposed by Rosellini *et al*. for circular disks and approximated the wetted area as a circular disk with radius *λ*_*eq*_*R*, modeling the hydrodynamic force as

1 $$F=\frac{1}{2}{\rho }_{w}|{U}_{B}{|}^{2}{S}_{w}\,\sin (\alpha +{\beta }_{B}),$$

where *S*_*w*_ is the sphere wetted area. Their published results show that this model predicts the skipping behavior of an elastomeric sphere in terms of impact velocity *U*_0_ and impact angle *β*_0_ over four subsequent skipping events. However, their paper does not comment on the ability of the model to predict impact force, number of impact events or distance travelled^13^.

We present two cases, which were characteristic of observed traditional skipping and water walking, and compare the results with model predictions. For each scenario, experimentally measured impact conditions *U*_0,1_ and *β*_0,1_ (the second subscript refers to the impact number) are used to calculate exit conditions *U*_*E*,1_ and *β*_*E*,1_. Values of *U*_*E*,*N*_ are calculated from the analytical model, and values of *β*_*E*,*N*_ are calculated from a fit to empirical data. These exit conditions were entered into a ballistic model with simplified air drag to calculate the distance of flight and generate values for *U*_0,2_ and *β*_0,2_. The process was repeated until the impact angle ${\beta }_{0,N} < 0.001^\circ$, at which point the impact was deemed an entry. In this way values for *U*_0_, *U*_*E*_, *β*_0_, *β*_*E*_ and the acceleration magnitude *a* were modeled for each impact, in addition to predictions for number of skips *N* and total distance *d* for each complete skipping event.

**Figure 4:**
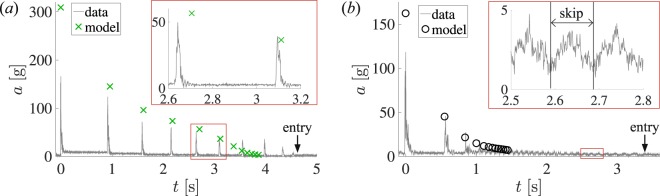
(**a**) The acceleration magnitude *a* is plotted for a traditional skipping event (gray lines). The peak acceleration values *a* from our skipping model with the same initial conditions as the experimental first skip are marked with green × marks. The model does not predict the exact same magnitude or peak times, but is relatively close (inset). (**b**) The acceleration magnitude *a* is plotted for a water walking skipping event (gray lines). The peak acceleration values from our skipping model with the same initial conditions as the experimental first skip are indicated with black $\circ$ marks. The model doesn’t skip as many times as the experiment (inset). In each of the plots above, the events are aligned in time at the first impact event. The accelerometer signal to noise ratio is 57.3.

For the traditional skipping case, a sphere with radius $R=0.05\,{\rm{m}}$, material shear modulus $G=74.4\,{\rm{kPa}}$, specific gravity $SG=1.07$ and containing a small 6-axis accelerometer was shot toward the water free surface with impact conditions of ${\beta }_{0}=10.7^\circ$ and ${U}_{0}=45.1$ m/s. The sphere skipped in a traditional manner $N=10$ times and traveled $d=105\,{\rm{m}}$ before entry. Figure 4(a) presents the acceleration magnitude *a* for this skipping event (gray lines). The measured acceleration peaks decrease relatively slowly, then decay more quickly between the final and more rapid impacts of “pitty-pat” prior to entry. The impacts in the middle of the skipping event (highlighted in the plot inset) have a magnitude near $a=50\,g$ with well-defined peaks. The peak values of *a* for a modeled skipping event with the same initial impact conditions are plotted on the same graph (green marks). The timeline for the modeled and experimental data are aligned at the first impact event. For this case, the model significantly over predicts *a* for the initial impact, but shows good agreement through the sixth impact event, predicting $N=12$, $d=83\,{\rm{m}}$ (*measured:* $N=10$, $d=105\,{\rm{m}}$) and also accurately predicts a “pitty-pat” event prior to entry. In the model, the impacting object is represented by a disk rather than an oblate spheroid, resulting is an overestimation of the impact forces.

In the water walking case, a sphere with $R=0.05\,{\rm{m}}$, $G=12.3\,{\rm{kPa}}$, $SG=1.07$ and containing a small 6-axis accelerometer was shot toward the water free surface with impact conditions of ${\beta }_{0}=11.8^\circ$ and ${U}_{0}=29.2$ m/s. The sphere skipped in a traditional manner three times before the vertical motion of the sphere all but ceased and the sphere traversed nearly parallel to the water surface. During this time the sphere was striking the water so frequently the splash crowns commonly overlapped. The splash events are so intermingled that it is impossible to count the number of impact events visually from images with confidence. However, individual impact events can be identified via small peaks in the accelerometer data (Fig. 4(b) inset). The experimental sphere was observed to skip $N=30$ times over a distance of $d=52\,{\rm{m}}$. The measured and calculated accelerations are shown in Fig. 4(b), where we see the peak *a* values decreasing more quickly than for the skipping case shown in Fig. 4(a). The peak values of *a* from a simulated skipping event with the same initial impact conditions for the first skip are plotted on the same graph in Fig. 4(b) (black marks). Again the model significantly over predicts *a* for the initial impact, but shows good agreement through the seventh impact event, predicting $N=13$ and $d=24\,{\rm{m}}$ (*measured:* $N=30$, $d=52\,{\rm{m}}$). The model accurately predicts a transition to rapid, low acceleration impact events between 3–4 skips, which enables the transition to water walking. However, the model significantly under-predicts both number of skips *N* and distance travelled *d* for walking walking cases. This hints at the fact that the dynamics are sufficiently different in this water walking mode compared to traditional skipping, which is well captured by the model.

**Figure 5:**
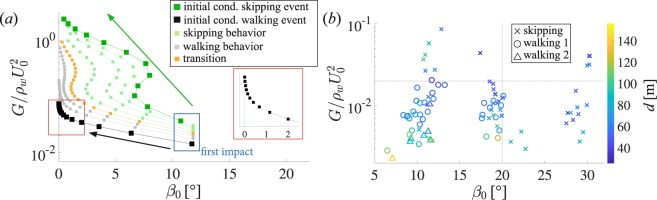
(**a**) Skipping of elastomeric spheres can be predicted from the dimensionless terms *G*/${\rho }_{w}{U}_{0}^{2}$ and *β*_0_. A modeled skipping event, with the same initial conditions as the traditional case shown in Fig. 4(a), is marked by dark green squares. The event starts with the bottom-right marker and progresses in the direction of the green arrow. A modeled skipping event, with the same initial conditions as the water walking case shown in Fig. 4(b), is marked by black squares. A close up view of the final skips is presented in the inset. The skipping model does not distinguish between skipping types, rather, it can only indicate whether a skip occurred or not, thus the model symbols are constant throughout the plot. Six additional skipping events were modeled, with initial conditions ranging from the traditional skipping event to the water walking event, and are marked on the plot with smaller squares. Light green squares exhibit traditional skipping characteristics, gray squares exhibit water walking characteristics and orange squares exhibit mixed characteristics, or a transition (**b**) The initial impact conditions for every experimental multi-skip event analyzed in this study are plotted in the same parameter space. A traditional skip is marked by ×, where water walking skipping events are marked by $\circ$ and Δ (differentiated later). Water walking was only observed when *G*/${\rho }_{w}{U}_{0}^{2} < 0.02$ and ${\beta }_{0} < 20^\circ$, indicated by dotted gray lines. The orange skipping event in (**a**) had an initial value of *G*/${\rho }_{w}{U}_{0}^{2}=0.02$, corresponding to the horizontal dotted gray line in (**b**). The color of the symbols denotes the distance the sphere traveled before entry *d*, as indicated by the colored sidebar.

Research by Belden *et al*.^13^ showed that for elastomeric spheres with specific gravity near unity ($SG\approx 1$), skipping or entry can be predicted from two dimensionless parameters: *G*/${\rho }_{w}{U}_{0}^{2}$ and *β*_0_. In Fig. 5(a) *G*/${\rho }_{w}{U}_{0}^{2}$ is plotted against *β*_0_ for the two mathematically modeled skipping events (i.e., traditional and water-walking) introduced in Fig. 4. The calculations for the traditional skipping event are plotted with dark green squares in Fig. 5(a). Impact angle *β*_0_ and impact velocity *U*_0_ generally decrease over the skipping event, which ends in water entry (upper-most green square). The modeled water-walking case (black squares) exhibits markedly different behavior. Impact angle *β*_0_ decreases rapidly over the first 3–4 skips (transition to water walking was always observed to occur within 3–4 skips in experiments). Impact velocity *U*_0_ does not decrease as rapidly as the traditional skipping case. The model then predicts several successive impact events with very low values of *β*_0_, yet small changes in *G*/${\rho }_{w}{U}_{0}^{2}$ (impact velocity). In other words the model predicts a quick transition to impact events which remain close to the water surface (low impact angles), with minimal change in velocity magnitude, which is characteristic of water walking. The smaller squares represent model-generated skipping events with initial conditions between the walking behavior and the traditional skipping behavior. As *G*/${\rho }_{w}{U}_{0}^{2}$ increases, the modeled skipping behavior transitions from characteristics of a water walking event to a traditional skipping event.

Figure 5(b) presents the placement of the initial skip on the regime diagram for all experimental multi-skip events analyzed for this study. Traditional skipping events are indicated by × marks where water walking is indicated with $\circ$ and Δ marks (differentiated later). Note that water walking was only observed when *G*/${\rho }_{w}{U}_{0}^{2} < 0.02$ and ${\beta }_{0} < 20^\circ$ (gray dotted lines). The largest values for *d* were observed when *G*/${\rho }_{w}{U}_{0}^{2}$ and *β*_0_ were minimized. Based on the experimentally determined transition value from Fig. 5(b), we apply these initial conditions to a simulation and find the orange result in Fig. 5(a). This simulation qualitatively shows characteristics of the traditional and walking skip types and is thus consistent as a transition between these regimes.

### Investigating water walking

A primary difference we observed for water walking compared to traditional skipping is the presence of significant rigid body rotation with a corresponding angular velocity $\dot{\psi }$ that we observe to be relatively constant throughout the water walking portion of the skipping event. To investigate the role that rotation might play in water walking, we modify the skipping model presented Belden *et al*.^13^. They provide a set of equations for the motion of the center of mass (*d*_1_, *d*_2_) and sphere deformation (*λ*_1_, *λ*_2_, *λ*_3_, *α*) in terms of general forces and tractions acting on the body. Here, *λ*_1_ and *λ*_2_ are principal stretches describing the deformation of the sphere, as shown in Fig. 3, and by definition ${\lambda }_{3}=1$/${\lambda }_{1}{\lambda }_{2}$ to conserve volume. The principal stretches are aligned with principal axes that, in general, may be rotated by an angle *α* with respect to the world coordinate frame. We expand the version of the model given by Belden *et al*. to include rigid body rotation $\dot{\psi }$; Appendix A (Supplementary Information) contains a full derivation of the model, which yields a set of coupled second order nonlinear ordinary differential equations for *d*_1_, *d*_2_, *λ*_1_, *λ*_2_, *α* and now $\psi$. By looking for steady-state solutions with a constant rigid rotation rate $\dot{\psi }$, constant principal axes rotation rate $\dot{\alpha }$ and all other derivatives and tractions vanishing, the system reduces to two equations of motion relating $\dot{\psi }$ to the principal stretches *λ*_1_ and *λ*_2_,

2 $$\frac{4\pi }{15}{\rho }_{{\rm{s}}}{R}^{5}\,[\,-\,2{\dot{\alpha }}^{2}({\lambda }_{1}-{\lambda }_{2})+2\dot{\alpha }\dot{\psi }({\lambda }_{1}-{\lambda }_{2})-{\lambda }_{1}{\dot{\psi }}^{2}]+\frac{4\pi }{3}G{R}^{3}({\lambda }_{1}-\frac{{\lambda }_{2}}{{({\lambda }_{1}{\lambda }_{2})}^{3}})=0,$$

3 $$\frac{4\pi }{15}{\rho }_{{\rm{s}}}{R}^{5}\,[2{\dot{\alpha }}^{2}({\lambda }_{1}-{\lambda }_{2})-2\dot{\alpha }\dot{\psi }({\lambda }_{1}-{\lambda }_{2})-{\lambda }_{2}{\dot{\psi }}^{2}]+\frac{4\pi }{3}G{R}^{3}({\lambda }_{2}-\frac{{\lambda }_{1}}{{({\lambda }_{1}{\lambda }_{2})}^{3}})=0$$

Adding these equations results in

4 $$\frac{4\pi }{15}{\rho }_{{\rm{s}}}{R}^{5}\,[\,-\,{\dot{\psi }}^{2}({\lambda }_{1}+{\lambda }_{2})]+\frac{4\pi }{3}G{R}^{3}[{\lambda }_{1}+{\lambda }_{2}-\frac{{\lambda }_{1}}{{({\lambda }_{1}{\lambda }_{2})}^{3}}-\frac{{\lambda }_{2}}{{({\lambda }_{1}{\lambda }_{2})}^{3}}]=0$$

Solving for $\dot{\psi }$ gives

5 $${\dot{\psi }}^{2}=\frac{5G}{{\rho }_{s}{R}^{2}}(1-\frac{1}{{({\lambda }_{1}{\lambda }_{2})}^{3}})$$

where ${\rho }_{s}$ is the sphere density. Equation 2 has a family of real solutions requiring ${\lambda }_{1}\ge 1$/*λ*_2_. Rearranging equation 5 gives

6 $${\lambda }_{1}{\lambda }_{2}={(\frac{1}{1-{(\dot{\psi }/\sqrt{\frac{5G}{{\rho }_{s}{R}^{2}}})}^{2}})}^{\frac{1}{3}}.$$

**Figure 6:**
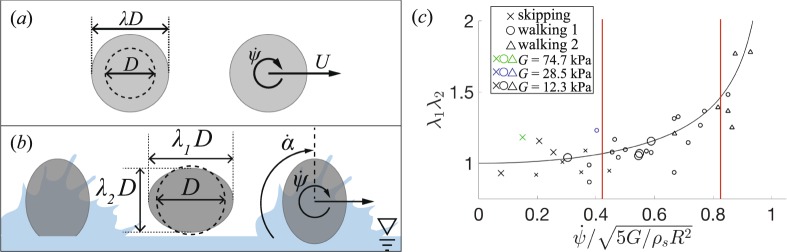
(**a**) Idealized water walking is diagramed with indications of the stretch parameter *λ* and angular velocity $\dot{\psi }$. (**b**) In observations of experimental skipping events *λ*_1_ and *λ*_1_ are generally not equal, creating a slightly oblong shape. The parameters *λ*_1_ and *λ*_1_ were measured from high speed images. (**c**) The stretch parameter *λ*_1_*λ*_2_ is plotted against dimensionless angular velocity $\dot{\psi }/\sqrt{5G/{\rho }_{s}{R}^{2}}$ for all measured multi-skip cases. The derived analytical expression (equation 6, black line) predicts that *λ*_1_*λ*_2_ increases exponentially with increasing $\dot{\psi }/\sqrt{5G/{\rho }_{s}{R}^{2}}$. Traditional skipping was not observed when $\dot{\psi }/\sqrt{5G/{\rho }_{s}{R}^{2}}\gtrsim 0.41$, which corresponds to $\dot{\psi }$/$\dot{\alpha }=0.5$. The transition from type 1 to type 2 water walking (defined in Fig. 7) occurs when $\dot{\psi }/\sqrt{5G/{\rho }_{s}{R}^{2}}\approx 0.83$, which corresponds to $\dot{\psi }$/$\dot{\alpha }=1$.

Thus equation 6 describes a sphere in steady-state translation and rotation that holds a constant deformed shape described by *λ*_1_*λ*_2_. This type of skipping event is represented visually in Fig. 6(a). The expression predicts higher values of *λ*_1_*λ*_2_ for larger values of $\dot{\psi }$, ${\rho }_{s}$ and *R* and for smaller values of *G*. The relationship in equation 6 is plotted as a black line in Fig. 6(c), which predicts an exponential increase in *λ*_1_*λ*_2_ as $\dot{\psi }/\sqrt{5G/{\rho }_{s}{R}^{2}}\to 1$. In observed experimental skipping events, *λ*_1_ and *λ*_2_ are not precisely equal, leading to a slightly oblong configuration as seen in Fig. 6(b). To compare theoretical predictions with observed behavior, the value of *λ*_1_*λ*_2_ is measured for several experimental cases by measuring the stretches in the plane of motion from high speed images. Angular velocity $\dot{\psi }$ is measured from high speed video as near to the middle of the skipping event as possible. These experimentally measured values of *λ*_1_*λ*_2_ are plotted against dimensionless angular velocity for several experimental cases in Fig. 6(c) and follow the predicted trend.

Belden *et al*.^13^ showed that natural elastic wave modes excited in the sphere propagate with angular velocity $\dot{\alpha }\sim \sqrt{5G/{\rho }_{s}{R}^{2}}$, which appears in the denominator of equation 6. The above analysis allows for arbitrary values of $\dot{\alpha }$, and we expect that elastic waves excited during water impact do indeed propagate within the sphere during water walking skip types. Figure 6(b) shows the kinematics observed in many water walking instances in the field. The sphere assumes a somewhat prolate shape with *λ*_1_ > *λ*_2_ and rotates with nearly constant rigid rotation rate $\dot{\psi }$. While difficult to experimentally identify the elastic waves given high sphere rotation, the impact conditions are consistent with mode 1^+^ elastic waves, which would tend to distort the sphere into the prolate shape^13^. It is thus interesting to consider the ratio ${\rm{\Omega }}\equiv \dot{\psi }/\sqrt{5G/{\rho }_{s}{R}^{2}}$ in terms of the rigid body rotation rate and the angular velocity of propagating elastic waves, $\dot{\alpha }$. Empirical results from Belden *et al*.^13^ showed that $\dot{\alpha }=0.83\sqrt{5G/{\rho }_{s}{R}^{2}}$ for mode 1^+^ elastic waves; substituting this into the expression for ${\rm{\Omega }}$ gives $\dot{\psi }/\dot{\alpha }={\rm{\Omega }}/0.83$. The vertical lines on Fig. 6(c) correspond to $\dot{\psi }/\dot{\alpha }=0.5$ and $\dot{\psi }/\dot{\alpha }=1$, and provide boundaries between three distinct skipping types: traditional skipping, water walking type 1 and water walking type 2. The difference between the two types of walking modes, and their relation to $\dot{\psi }$/$\dot{\alpha }$ is explored next.

**Figure 7:**
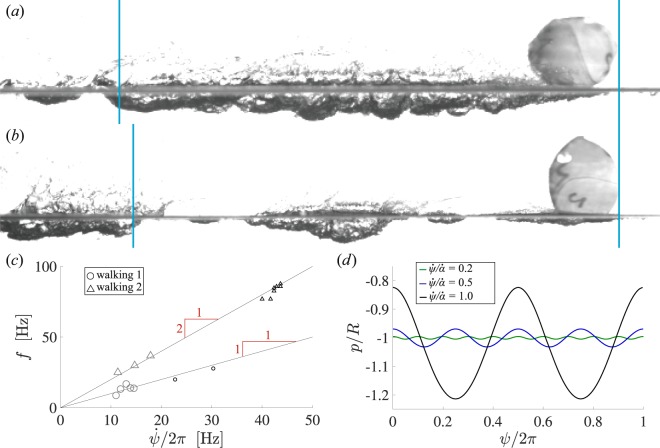
(**a**) Water walking spheres that skip approximately one time per rotation, such as the case shown here, are denoted as type 1 water walking. Cases that skip approximately two times per rotation, such as the case shown in (**b**), are denoted as type 2 water walking. Vertical blue lines indicate the distance over which a single rotation occurred. (Pertinent parameters; (**a**) *f* = 20.0 Hz, $\dot{\omega }$ = 22.7 rot/s = 142.8 rad/s, $\dot{\alpha }$ = 241.7 rad/s, $\dot{\omega }$/$\dot{\alpha }$ = 0.093; (**b**) *f* = 76.9 Hz, $\dot{\omega }$ 40.0 rot/s = 251.3 rad/s, $\dot{\alpha }$ = 241.7 rad/s, $\dot{\omega }$/$\dot{\alpha }$ = 1.04) (**c**) Impact events per second *f* are plotted against rotations per second $\dot{\psi }$/2*π*. For gray markers, *f* was measured from accelerometer data, where black markers indicate that *f* was measured from photographs. All water walking events fall on one of two slopes, indicating 1 or 2 impacts per sphere rotation. (**d**) The lowest point *p* in the **e**_**1**_-**e**_**2**_ plane on a theoretical sphere experiencing a rigid rotation and volume-preserving stretch is plotted as a function of rotation angle for a single rotation. When the ratio of rotation rate to angular wave speed is relatively small ($\dot{\psi }$/$\dot{\alpha }=0.2$), the variation in *p*/*R* is relatively small (green line). At $\dot{\psi }$/$\dot{\alpha }=0.5$ the difference between the low points become more significant (blue line). The number of peaks approaches 2 as $\dot{\psi }$/$\dot{\alpha }\to 1$.

Figure 7(c) plots the frequency of impact events *f* as a function of $\dot{\psi }$, looking at only water walking cases (For traditional skipping events, the time between impact events decreased dramatically with each impact and did not correlate with $\dot{\psi }$). Black markers indicate events where skips could be counted visually. Gray markers indicate events where skips were measured from accelerometer data. The observed water walking can be distinguished by one of two slopes, experiencing impact events approximately once or twice per sphere rotation. In Fig. 7(a) a sphere is shown along with the length over which a single rotation occurred (vertical blue lines). Note that only a single cavity was formed; this type of water walking is identified as type 1 water walking. By contrast, the sphere shown in Fig. 7(b) impacts the free surface two times over a single sphere rotation, exhibiting what we call type 2 water walking. Note that in Fig. 5(b), water walking type 2 only occurs at the smallest values of *G*/${\rho }_{w}{U}_{0}^{2}$ and *β*_0_.

We have discussed that the coupling between $\dot{\psi }$ and $\dot{\alpha }$ play an important role in predicting water walking behavior. We seek additional insight into this observation by considering the position of a single point **x** on the outer surface of the sphere that is constrained to the **e**_**1**_-**e**_**2**_ plane such that ${\bf{x}}=R\,\cos \,\gamma {{\bf{e}}}_{{\bf{1}}}+R\,\sin \,\gamma {{\bf{e}}}_{{\bf{2}}}$, where *γ* is a parameter. We assume that the sphere undergoes a rigid rotation through angle $\psi (t)$ and a volume preserving stretch defined by *λ*_1_, *λ*_2_ and *α*(*t*) (see Appendix A, Supplementary Information). The **e**_**2**_ component of **x** is differentiated to solve for the value of *γ* corresponding to the lowest point on the sphere, *p*(*t*). Figure 7(c) plots *p*(*t*) over a full period of rigid rotation for different ratios of $\dot{\psi }$/$\dot{\alpha }$.

For small values of $\dot{\psi }$/$\dot{\alpha }$, deformation is minimal and the lowest point of the sphere, *p*(*t*), varies little over a single rotation (Fig. 7(c)),which would produce only small scale disturbances on the cavity surface. At the other extreme, when $\dot{\psi }$/$\dot{\alpha }$ = 1, the amplitude of *p*(*t*) is significant and there are two large valleys in one period of rigid rotation. This corresponds to the onset of water walking type 2, in which two distinct cavities are formed in one sphere rotation period (as shown in Fig. 7(c)). For $0.5 < \dot{\psi }$/$\dot{\alpha } < 1$, the amplitude of *p*(*t*) is smaller and the frequency is larger. We would therefore expect smaller scale and more frequent disturbances to appear on the cavity, which is supported by Fig. 7(a). This range of $\dot{\psi }$/$\dot{\alpha }$ coincides with one contiguous cavity appearing for each rotation period consistent with type 1 water walking.

## Discussion

To extend our knowledge of elastomeric spheres skipping a single time to multi-skip events, spheres of varying sizes and shear moduli *G* are propelled across a lake surface with a wide range of impact conditions (impact velocity *U*_0_ and impact angle *β*_0_). These skipping events are captured and quantified using high speed cameras, low speed cameras and image processing; the results are compared with mathematically modeled multi-skip events. For traditional skipping, the model exhibits accurate estimation of impact velocity *U*_0_, impact angle *β*_0_ and peak acceleration magnitude *a*. For the representative cases shown, the model provides a reasonable prediction for number of skips *N* and distance travelled *d*.

This large-scale experimental effort led to the discovery of a new skipping mode for elastomeric spheres labeled “water walking”, which is characterized by a sphere with comparatively high angular velocity $\dot{\psi }$ maintaining a constant deformed prolate posture. It also involves the sphere moving nearly parallel to the water surface while the tips of the major axis of the spheroid impact the water surface rapidly. Simulated skipping events with the same initial conditions as water walking predict comparable values for *a*, *U*_0_ and *β*_0_, but underestimate *N* and *d*. However, the model does predict a transition to similar impact events with very low values of *β*_0_, which is characteristic of the transition to water walking.

Water walking occurs when the sphere acquires significant rigid body rotation, which incites a persistent, deformed sphere shape. The amount of rotation necessary varies with sphere shear modulus, radius and density. Experiments have shown this deformed shape to resemble a prolate spheroid, which indicates that the sphere is not just undergoing a rigid body rotation but also has elastic waves propagating within it. The ratio of rigid rotation rate to the angular rate of the elastic waves classifies the type of water walking observed. In type 2 walking, the tips of the spheroid dip below the surface approximately twice per rotation cycle creating two distinct cavities. For type 1 walking, the amplitude of perturbations in the position of the lowest point on the sphere is smaller than for type 2 walking. As a result, the lowest point doesn’t repeatedly rise above the static free-surface level as in the type 2 case. Instead, approximately one contiguous cavity is formed in each rotation cycle, although the cavity surface is wavy due to interactions with the deforming sphere.

Our results show no observed correlation between number of skips *N* and the distance of the skipping event *d* (See Appendix B, Supplementary Information). However, water walking events consistently produce values of *N* that are an order of magnitude higher than traditional skipping events.

## Methods

We investigated the water skipping characteristics of deformable elastomeric spheres by creating multi-skip events on a lake surface (Bear Lake, UT, USA). Data were only collected during the early morning or early evening hours when the lake was smooth and waves were minimal (see Fig. 1). We varied sphere impact velocity *U*_0_, impact angle *β*_0_, diameter *D* and material shear modulus *G*. Spheres were made from a platinum-cure silicone rubber called Dragon Skin^®^, produced by Smooth-On, Inc. Sphere shear modulus *G* was altered by adding a silicone thinner to the mixture to produce three values (*G* = 12.3, 28.5 & 74.7 kPa) with a density of ${\rho }_{s}=1070$ kg/m^3^. The ingredients were measured by mass ratio, then mixed and placed into a vacuum chamber to remove entrained air. The resulting mixtures were poured into aluminum molds to form spheres of two diameters (*R* = 25.5 & 50 mm). Prior research shows that these spheres can be reasonably modeled as neo-Hookean solids^13^.

**Figure 8:**
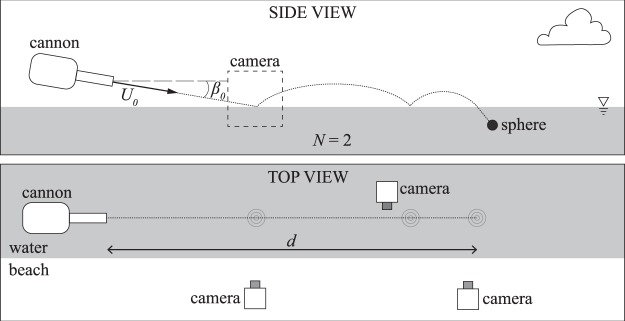
Experimental spheres of radius *R* and shear modulus *G* were propelled from a pneumatic air cannon toward a lake surface. The cannon allowed for adjustment to impact velocity *U*_0_ and impact angle *β*_0_. The initial impact event was filmed with a high speed camera, while subsequent impact events were filmed with low and high speed cameras. The total distance travelled before entry is represented by *d*; *N* represents the number of skips before entry.

Spheres were launched from a pneumatic cannon at the water surface to initiate skipping, as shown in Fig. 8. Air pressure was varied to control *U*_0_ and the barrel angle was adjusted to alter *β*_0_. Impact conditions were filmed using a Photron SA3 high speed camera filming at 1000 fps; this footage was used to measure *U*_0_ and *β*_0_. Downrange footage was captured using several different cameras (Photron SA3, Sony A7R, GoPro and iPhone). This experimental setup on the lakeside is shown in Fig. 8. A six-axis ±200 *g* accelerometer was embedded within some of the larger spheres (*R* = 50 mm) to measure acceleration during skipping. The number of times a sphere skips before entry is indicated by *N*, and was measured from high speed video. The distance from the cannon barrel tip to water entry (end of skipping) is denoted by *d*, and was measured by eye using markers along the shore.

Additional experiments were conducted in a 0.9 × 1.2 × 27.4 m tank. The skipping events were filmed with a Phantom v310 high speed camera using diffuse backlighting.

## Supplementary information

Supplementary Information

## Data Availability

The datasets generated and analysed during the current study are available from the corresponding author on reasonable request.
