# Supplementary material for: Water walking as a new mode of free surface skipping
Source: Sci Rep. 2019 Apr 15;9:6042. doi: 10.1038/s41598-019-42453-x (PMC6465409; doi:10.1038/s41598-019-42453-x)
Supplement: Supplementary file 1 — Supplementary Information [file 41598_2019_42453_MOESM1_ESM.pdf]

Supplementary Information
Water walking as a new mode of free surface skipping

Randy C. Hurd[1,*], Jesse Belden[2], Allan F. Bower[3], Sean Holekamp[2], Michael A. Jandron[2], and Tadd T. Truscott[1]

[1]Utah State University, Department of Mechanical & Aerospace Engineering, Logan, UT 84321, USA
[2]Naval Undersea Warfare Center, Newport, RI 02841, USA
[3]Brown University, School of Engineering, Providence, RI 02912, USA
[*]randyhurd@gmail.com

ABSTRACT

Appendix A. Modeling water walking behavior

A.1. General model of sphere deformation

This section derives an approximate analytical approach to modeling the impact between a compliant elastomeric sphere and a fluid surface. The goal is to describe the deformation and motion using a set of reduced, scalar generalized coordinates (to be outlined) which are governed by a system of ordinary differential equations (ODEs). We begin by defining a fixed Cartesian coordinate system with $\mathbf{e_1}$ parallel to the horizontal component of velocity of the sphere, $\mathbf{e_2}$ perpendicular to the surface of the fluid, and $\mathbf{e_3}$ transverse to the motion (see Fig. 5 in paper).

We assume that the sphere is a homogeneous, isotropic, incompressible neo-Hookean solid with radius R, shear modulus G and mass density ρ_s. The sphere moves parallel to the $\mathbf{e_1}$ direction and rotates only about the $\mathbf{e_3}$ axis. The deformation of the sphere is then described as a sequence of three deformations:

1. A rigid displacement through a vector $\mathbf{d}$ that describes the motion of the center of mass (COM). The initial conditions, momentum conservation and symmetry require that $d_3 = 0$ throughout the motion.
2. A rigid rotation $\mathbf{Q}$ through an angle ψ about the $\mathbf{e_3}$ axis (this does not change the shape of the solid).
3. A volume preserving stretch $\mathbf{V}$ that deforms the sphere into an ellipsoid. By symmetry, one of the principal directions of stretch must be parallel to the $\mathbf{e_3}$ direction. The other two principal stretch directions are parallel to unit vectors $\mathbf{m_1}, \mathbf{m_2}$, which lie in the vertical plane (see Fig. 5 and Fig. A1). We let α be the angle between the $\mathbf{m_1}$ and $\mathbf{e_1}$ directions (positive α corresponds to rotation of the principal stretch directions about the $\mathbf{m_3}$ axis); thus α describes the attack angle of the ellipsoid. We introduce the principal stretches λ_1, λ_2 and $\lambda_3 = 1/\lambda_1\lambda_2$. The coordinates of a material particle in the sphere before deformation are given by $\mathbf{x}$, and $\mathbf{y}$ defines the coordinates after deformation.

The following identities are useful for further calculations. Elementary trigonometry shows that the unit vectors $\mathbf{e}_i$ and $\mathbf{m}_i$ are related by

$$\mathbf{e}_i = \mathbf{P}\mathbf{m}_i \tag{1}$$

where $\mathbf{P}$ is a proper orthogonal tensor whose components in both the basis $\{\mathbf{e_1}, \mathbf{e_2}, \mathbf{e_3}\}$ and $\{\mathbf{m_1}, \mathbf{m_2}, \mathbf{m_3}\}$ can be expressed as a matrix

$$[\mathbf{P}] = \begin{bmatrix} \cos\alpha & -\sin\alpha & 0 \\ \sin\alpha & \cos\alpha & 0 \\ 0 & 0 & 1 \end{bmatrix} \tag{2}$$

Since $\mathbf{e}_i$ are independent of time, derivatives of the vectors $\mathbf{m}_i$ can be calculated as

$$\dot{\mathbf{m}}_i = \frac{d}{dt}\mathbf{P}^T\mathbf{e}_i \tag{3}$$

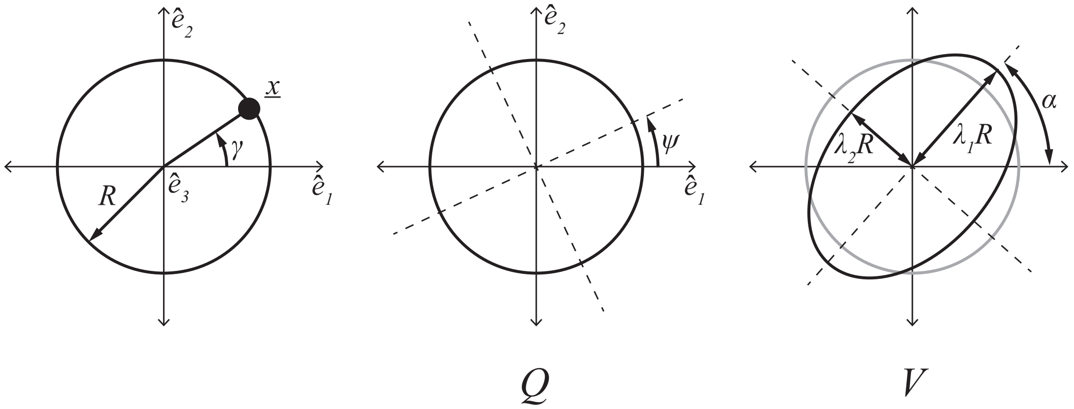

Figure 1. Two-dimensional depiction of the sphere in the undeformed and deformed state. (Left) The location of a point $\mathbf{x}$ on the $\mathbf{e_1}-\mathbf{e_2}$ plane of the undeformed sphere is described by the angle γ. (Middle) The sphere undergoes a rigid body rotation $\mathbf{Q}$ through an angle ψ about the $\mathbf{e_3}$ axis that does not change the shape of the solid. (Right) The sphere undergoes a volume preserving stretch $\mathbf{V}$ that deforms the sphere into an ellipsoid. The principal stretches are defined as λ_1, λ_2 and $\lambda_3 = 1/\lambda_1\lambda_2$ and we let α be the angle between the $\mathbf{m_1}$ and $\mathbf{e_1}$ directions, positive α corresponds to rotation of the principal stretch directions about the $\mathbf{m_3}$ axis.

Since $\mathbf{P}$ is orthogonal it follows that

$$\mathbf{P}\mathbf{P}^T = \mathbf{I} \implies \frac{d\mathbf{P}}{dt}\mathbf{P}^T + \mathbf{P}\frac{d\mathbf{P}^T}{dt} = 0 \implies \frac{d\mathbf{P}^T}{dt} = \mathbf{P}^T\frac{d\mathbf{P}}{dt}\mathbf{P}^T \tag{4}$$

Substituting this result into equation 3 and making use of equation 1 yields

$$\dot{\mathbf{m}}_i = \mathbf{P}^T\frac{d\mathbf{P}}{dt}\mathbf{m}_i \tag{5}$$

Evaluating this expression shows that

$$\begin{aligned} \dot{\mathbf{m}}_\mathbf{1} &= \dot{\alpha}\mathbf{m_2} \\ \dot{\mathbf{m}}_\mathbf{2} &= -\dot{\alpha}\mathbf{m_1} \\ \dot{\mathbf{m}}_\mathbf{3} &= 0 \end{aligned} \tag{6}$$

The standard definition of principal stretches implies that the volume preserving stretch $\mathbf{V}$ can be expressed as

$$\mathbf{V} = \lambda_1\mathbf{m_1}\otimes\mathbf{m_1} + \lambda_2\mathbf{m_2}\otimes\mathbf{m_2} + \frac{1}{\lambda_1\lambda_2}\mathbf{m_3}\otimes\mathbf{m_3} \tag{7}$$

where $\mathbf{a}\otimes\mathbf{b}$ denotes the tensor product of two vectors; i.e the operator with the property that $[\mathbf{a}\otimes\mathbf{b}]\cdot\mathbf{c} = (\mathbf{b}\cdot\mathbf{c})\,\mathbf{a}$ for all vectors $\mathbf{c}$. Taking the time derivative of this expression and using equation 6 then shows that

$$\begin{aligned} \dot{\mathbf{V}} &= \dot{\lambda}_1\mathbf{m_1}\otimes\mathbf{m_1} - \frac{\dot{\lambda}_1\lambda_2 + \lambda_1\dot{\lambda}_2}{(\lambda_1\lambda_2)^2}\mathbf{m_3}\otimes\mathbf{m_3} + \dot{\lambda}_2\mathbf{m_2}\otimes\mathbf{m_2} \\ &+ (\lambda_1-\lambda_2)\,\dot{\alpha}\,(\mathbf{m_2}\otimes\mathbf{m_1} + \mathbf{m_1}\otimes\mathbf{m_2}) \end{aligned} \tag{8}$$

and

$$\ddot{\mathbf{V}} = \ddot{\lambda}_1 \mathbf{m_1} \otimes \mathbf{m_1} - \frac{\ddot{\lambda}_1 \lambda_2 + \lambda_1 \ddot{\lambda}_2}{(\lambda_1 \lambda_2)^2} \mathbf{m_3} \otimes \mathbf{m_3} + \ddot{\lambda}_2 \mathbf{m_2} \otimes \mathbf{m_2} + (\lambda_1 - \lambda_2)\, \ddot{\alpha} \,(\mathbf{m_2} \otimes \mathbf{m_1} + \mathbf{m_1} \otimes \mathbf{m_2})$$
$$+ \left(\frac{2\left(\dot{\lambda}_1 \lambda_2 + \lambda_1 \dot{\lambda}_2\right)^2}{(\lambda_1 \lambda_2)^3} - \frac{2\dot{\lambda}_1 \dot{\lambda}_2}{(\lambda_1 \lambda_2)^2} \right) \mathbf{m_3} \otimes \mathbf{m_3} - 2(\lambda_1 - \lambda_2)\, \dot{\alpha}^2 (\mathbf{m_1} \otimes \mathbf{m_1} - \mathbf{m_2} \otimes \mathbf{m_2})$$
$$+ \left(\dot{\lambda}_1 - \dot{\lambda}_2\right) \dot{\alpha} \,(\mathbf{m_2} \otimes \mathbf{m_1} + \mathbf{m_1} \otimes \mathbf{m_2}) \tag{9}$$

The following identities are useful for further calculations:

$$\begin{aligned}
\mathbf{Q} &= \cos\psi\,(\mathbf{m_1} \otimes \mathbf{m_1} + \mathbf{m_2} \otimes \mathbf{m_2}) + \mathbf{m_3} \otimes \mathbf{m_3} - \sin\psi\,(\mathbf{m_1} \otimes \mathbf{m_2} - \mathbf{m_2} \otimes \mathbf{m_1}) \\
\dot{\mathbf{Q}} &= -\dot{\psi} \sin\psi\,(\mathbf{m_1} \otimes \mathbf{m_1} + \mathbf{m_2} \otimes \mathbf{m_2}) - \dot{\psi} \cos\psi\,(\mathbf{m_1} \otimes \mathbf{m_2} - \mathbf{m_2} \otimes \mathbf{m_1}) \\
\ddot{\mathbf{Q}} &= -\left(\ddot{\psi} \sin\psi + \dot{\psi}^2 \cos\psi\right)(\mathbf{m_1} \otimes \mathbf{m_1} + \mathbf{m_2} \otimes \mathbf{m_2}) \\
&- \left(\ddot{\psi} \cos\psi - \dot{\psi}^2 \sin\psi\right)(\mathbf{m_1} \otimes \mathbf{m_2} - \mathbf{m_2} \otimes \mathbf{m_1}) \\
\dot{\mathbf{Q}}\mathbf{Q}^T &= -\dot{\psi}\,(\mathbf{m_1} \otimes \mathbf{m_2} - \mathbf{m_2} \otimes \mathbf{m_1}) \\
\ddot{\mathbf{Q}}\mathbf{Q}^T &= -\dot{\psi}^2 (\mathbf{m_1} \otimes \mathbf{m_1} + \mathbf{m_2} \otimes \mathbf{m_2}) - \ddot{\psi}\,(\mathbf{m_1} \otimes \mathbf{m_2} - \mathbf{m_2} \otimes \mathbf{m_1})
\end{aligned} \tag{10}$$

The coordinates d_1, d_2 and ψ describe the rigid body motion and λ_1, λ_2 and α describe the deformation of the sphere. Our goal is to calculate equations of motion for these generalized coordinates. With this description we can write the deformation mapping as

$$\mathbf{y} = \mathbf{d} + \mathbf{V}\mathbf{Q}\mathbf{x} \tag{11}$$

The velocity and acceleration fields follow as

$$\mathbf{v} = \dot{\mathbf{d}} + \left(\dot{\mathbf{V}}\mathbf{Q} + \mathbf{V}\dot{\mathbf{Q}}\right)\mathbf{x} \tag{12}$$
$$\mathbf{a} = \ddot{\mathbf{d}} + \left(\ddot{\mathbf{V}}\mathbf{Q} + 2\dot{\mathbf{V}}\dot{\mathbf{Q}} + \mathbf{V}\ddot{\mathbf{Q}}\right)\mathbf{x} \tag{13}$$

We introduce a virtual velocity field

$$\delta\mathbf{v} = \delta\dot{\mathbf{d}} + \left(\delta\dot{\mathbf{V}}\mathbf{Q} + \mathbf{V}\delta\dot{\mathbf{Q}}\right)\mathbf{x} \tag{14}$$

where the kinematic variables are associated with virtual rates of change $\delta\dot{d}_1$, $\delta\dot{d}_2$, $\delta\dot{\psi}$, $\delta\dot{\lambda}_1$, $\delta\dot{\lambda}_2$, $\delta\dot{\alpha}$ about the current (deformed) state. The governing equations for d_1, d_2, ψ, λ_1, λ_2 and α are obtained from the principle of virtual work (i.e., weak form of the momentum conservation equation,[1]).

$$\int_V (\sigma : \delta\mathbf{D})dV + \int_V \rho_s(\mathbf{a} \cdot \delta\mathbf{v})dV + \int_V \rho_s(\mathbf{b} \cdot \delta\mathbf{v})dV - \int_A (\mathbf{t} \cdot \delta\mathbf{v})dA = 0 \tag{15}$$

or, in index notation,

$$\int_V \sigma_{ij}\delta D_{ij} dV + \int_V \rho_s a_i \delta v_i dV + \int_V \rho_s b_i \delta v_i dV - \int_A t_i \delta v_i dA = 0 \tag{16}$$

where b_i represents body forces, t_i are traction forces (i.e., applied to the sphere boundary) and V and A denote integration over the volume and surface of the deformed solid, respectively. The stretch rate in the solid is given by

$$D_{ij} = \frac{1}{2}\left(\frac{\partial v_i}{\partial y_j} + \frac{\partial v_j}{\partial y_i}\right) \tag{17}$$

The first term in equation 16 is the virtual rate of change of strain energy in the sphere, which can be calculated directly as

$$\int_V \sigma_{ij}\delta D_{ij}dV = \frac{4\pi}{3}GR^3\left(\lambda_1\delta\dot{\lambda}_1+\lambda_2\delta\dot{\lambda}_2-\frac{\lambda_1\delta\dot{\lambda}_2+\lambda_2\delta\dot{\lambda}_1}{(\lambda_1\lambda_2)^3}\right) \tag{18}$$

To evaluate the remaining terms, the following identities are useful

$$\begin{aligned}\int_{V_0} dV_0 &= \frac{4\pi}{3}R^3 \\ \int_{V_0} x_i dV_0 &= 0 \\ \int_{V_0} x_i x_j dV_0 &= \frac{4\pi}{15}R^5\delta_{ij}\end{aligned} \tag{19}$$

where x_i denote the coordinates of a material particle with respect to the center of the sphere and the integrals are evaluated over the undeformed sphere. Thus,

$$\int_V \rho_s b_i\delta v_i dV = \int_{V_0}\rho_s b_i\delta v_i dV_0 = -g\rho_s\frac{4\pi R^3}{3}\delta\dot{d}_2 \tag{20}$$

where we have made use of incompressibility to convert the integral over the volume of the deformed sphere (V) to an integral over the volume of the undeformed sphere (V_0). Also, we have noted that $b_i = -\rho_s g\delta_{i2}$, have substituted equation 14 for δv_i and have made use of the first two integrals in equation 19. Using equations 13-14 and again imposing incompressibility, the inertia term can be expressed as

$$\int_V \rho_s a_i\delta v_i dV = \int_{V_0}\rho_s a_i\delta v_i dV_0 = \int_{V_0}\rho_s\left[\ddot{\mathbf{d}}+\left(\ddot{\mathbf{V}}\mathbf{Q}+2\dot{\mathbf{V}}\dot{\mathbf{Q}}+\mathbf{V}\ddot{\mathbf{Q}}\right)\mathbf{x}\right]\cdot\left[\delta\dot{\mathbf{d}}+\left(\delta\dot{\mathbf{V}}\mathbf{Q}+\mathbf{V}\delta\dot{\mathbf{Q}}\right)\mathbf{x}\right]dV_0 \tag{21}$$

Expanding the terms on the right hand side yields

$$\begin{aligned}&\int_{V_0}\rho_s\ddot{\mathbf{d}}\delta\dot{\mathbf{d}}dV_0 = \frac{4\pi\rho_s R^3}{3}\left(\ddot{d}_1\delta\dot{d}_1+\ddot{d}_2\delta\dot{d}_2\right)\\ &\int_{V_0}\rho_s\left[\left(\ddot{\mathbf{V}}\mathbf{Q}+2\dot{\mathbf{V}}\dot{\mathbf{Q}}+\mathbf{V}\ddot{\mathbf{Q}}\right)\mathbf{x}\right]\cdot\delta\dot{\mathbf{d}}dV_0 = \int_{V_0}\rho_s\ddot{\mathbf{d}}\cdot\left[\left(\delta\dot{\mathbf{V}}\mathbf{Q}+\mathbf{V}\delta\dot{\mathbf{Q}}\right)\mathbf{x}\right]dV_0 = 0\\ &\int_{V_0}\rho_s\left[\left(\ddot{\mathbf{V}}\mathbf{Q}+2\dot{\mathbf{V}}\dot{\mathbf{Q}}+\mathbf{V}\ddot{\mathbf{Q}}\right)\mathbf{x}\right]\cdot\left[\left(\delta\dot{\mathbf{V}}\mathbf{Q}+\mathbf{V}\delta\dot{\mathbf{Q}}\right)\mathbf{x}\right]dV_0 =\\ &\frac{8\pi}{15}\rho_s R^5\left[\left(\ddot{\mathbf{V}}\mathbf{V}+2\dot{\mathbf{V}}\dot{\mathbf{Q}}\mathbf{Q}^T\mathbf{V}+\mathbf{V}\ddot{\mathbf{Q}}\mathbf{Q}^T\mathbf{V}\right):\delta\dot{\mathbf{V}}\mathbf{V}^{-1}+\left(\mathbf{V}\ddot{\mathbf{V}}+2\mathbf{V}\dot{\mathbf{V}}\dot{\mathbf{Q}}\mathbf{Q}^T+\mathbf{V}^2\ddot{\mathbf{Q}}\mathbf{Q}^T\right):\delta\dot{\mathbf{Q}}\mathbf{Q}^T\right]\end{aligned} \tag{22}$$

The nonzero terms can be interpreted physically as rates of change of translational and vibrational kinetic energies.

Finally, consider the term involving the external traction, which represents the pressure applied by the fluid on the elastomer surface,

$$\int_A t_i\delta v_i dA = \int_A \mathbf{t}\cdot\left[\delta\dot{\mathbf{d}}+\left(\delta\dot{\mathbf{V}}\mathbf{Q}+\mathbf{V}\delta\dot{\mathbf{Q}}\right)\mathbf{x}\right]dA \tag{23}$$

It is preferable to express the integrand in terms of spatial coordinates. Note that from equation 11 we have $\mathbf{x} = \mathbf{Q}^T\mathbf{V}^{-1}(\mathbf{y}-\mathbf{d})$ so that

$$\begin{aligned}\int_A t_i \delta v_i dA &= \int_A \mathbf{t}\cdot\left[\delta\dot{\mathbf{d}} + \left(\delta\dot{\mathbf{V}}\mathbf{Q} + \mathbf{V}\delta\dot{\mathbf{Q}}\right)\mathbf{Q}^T\mathbf{V}^{-1}(\mathbf{y}-\mathbf{d})\right]dA \\ &= \mathbf{F}\cdot\delta\dot{\mathbf{d}} + \left[\int_A \mathbf{t}\otimes(\mathbf{y}-\mathbf{d})\,dA\right] : \left[\delta\dot{\mathbf{V}}\mathbf{V}^{-1}\right] + \left[\int_A \mathbf{V}\mathbf{t}\otimes\mathbf{V}^{-1}(\mathbf{y}-\mathbf{d})\,dA\right] : \left[\delta\mathbf{Q}\cdot\mathbf{Q}^T\right]\end{aligned} \tag{24}$$

Here, $\mathbf{F}$ represents the resultant hydrodynamic force acting on the solid, the second term on the right hand side represents the virtual power associated with a force dipole tending to distort the elastomer and the third term on the right hand side represents a generalized moment (tending to make the body rotate).

Equation 16 can now be expressed in terms of the generalized coordinates. Substituting the expressions of equations 7-10 into equations 18,20-22 and 24, then setting each of $\delta\dot{d}_1$, $\delta\dot{d}_2$, $\delta\dot{\psi}$, $\delta\dot{\lambda}_1$, $\delta\dot{\lambda}_2$, $\delta\dot{\alpha}$ to be nonzero in turn will yield a set of coupled second order nonlinear ODEs. Working through this procedure yields the following governing equations:

$$\delta\dot{d}_1: \quad \frac{4\pi R^3}{3}\rho_s\ddot{d}_1 = F_h \tag{25}$$

$$\delta\dot{d}_2: \quad \frac{4\pi R^3}{3}\rho_s\ddot{d}_2 = F_v - \frac{4\pi R^3}{3}\rho_s g \tag{26}$$

$$\begin{aligned}\delta\dot{\psi}: \quad &\frac{4\pi}{15}\rho_s R^5\left[\ddot{\psi}\left(\lambda_1^2+\lambda_2^2\right) - \ddot{\alpha}\left(\lambda_1-\lambda_2\right)^2 + 2\dot{\psi}\left(\lambda_1\dot{\lambda}_1+\lambda_2\dot{\lambda}_2\right)\right] \\ &= \mathbf{m_3}\cdot\int_A\left(\mathbf{V^{-1}}(\mathbf{y}-\mathbf{d})\times\mathbf{V}\mathbf{t}\right)dA\end{aligned} \tag{27}$$

$$\begin{aligned}\delta\dot{\alpha}: \quad &\frac{4\pi}{15}\rho_s R^5\left[\left(2\ddot{\alpha}-\ddot{\psi}\right)\left(\lambda_1-\lambda_2\right)^2 + 2\dot{\psi}\left(\lambda_1-\lambda_2\right)\left(\dot{\lambda}_2-\dot{\lambda}_1\right)\right] \\ &= \left(\lambda_1-\lambda_2\right)\int_A\left(t_1\frac{(y_2-d_2)}{\lambda_2} + t_2\frac{(y_1-d_1)}{\lambda_1}\right)dA\end{aligned} \tag{28}$$

$$\begin{aligned}\delta\dot{\lambda}_1: \quad &\frac{4\pi}{15}\rho_s R^5\Bigg[\ddot{\lambda}_1\left(1+\frac{\lambda_2^2}{(\lambda_1\lambda_2)^4}\right) + \ddot{\lambda}_2\frac{\lambda_1\lambda_2}{(\lambda_1\lambda_2)^4} - 2\dot{\alpha}^2\left(\lambda_1-\lambda_2\right) + 2\frac{\lambda_2\dot{\lambda}_1\dot{\lambda}_2}{(\lambda_1\lambda_2)^4} \\ &-2\frac{\lambda_2\left(\dot{\lambda}_1\lambda_2+\lambda_1\dot{\lambda}_2\right)^2}{(\lambda_1\lambda_2)^5} + 2\dot{\alpha}\dot{\psi}\left(\lambda_1-\lambda_2\right) - \lambda_1\dot{\psi}^2\Bigg] + \frac{4\pi}{3}GR^3\left(\lambda_1 - \frac{\lambda_2}{(\lambda_1\lambda_2)^3}\right) \\ &= \int_A\left(t_1\frac{(y_1-d_1)}{\lambda_1} - t_3\frac{(y_3-d_3)}{\lambda_1}\right)dA\end{aligned} \tag{29}$$

$$\begin{aligned}\delta\dot{\lambda}_2: \quad &\frac{4\pi}{15}\rho_s R^5\Bigg[\ddot{\lambda}_2\left(1+\frac{\lambda_1^2}{(\lambda_1\lambda_2)^4}\right) + \ddot{\lambda}_1\frac{\lambda_1\lambda_2}{(\lambda_1\lambda_2)^4} - 2\dot{\alpha}^2\left(\lambda_2-\lambda_1\right) + 2\frac{\lambda_1\dot{\lambda}_1\dot{\lambda}_2}{(\lambda_1\lambda_2)^4} \\ &-2\frac{\lambda_1\left(\dot{\lambda}_1\lambda_2+\lambda_1\dot{\lambda}_2\right)^2}{(\lambda_1\lambda_2)^5} + 2\dot{\alpha}\dot{\psi}\left(\lambda_2-\lambda_1\right) - \lambda_2\dot{\psi}^2\Bigg] + \frac{4\pi}{3}GR^3\left(\lambda_2 - \frac{\lambda_1}{(\lambda_1\lambda_2)^3}\right) \\ &= \int_A\left(t_2\frac{(y_2-d_2)}{\lambda_2} - t_3\frac{(y_3-d_3)}{\lambda_2}\right)dA\end{aligned} \tag{30}$$

where F_h and F_v are the horizontal and vertical force components in $\{\mathbf{e_1},\mathbf{e_2},\mathbf{e_3}\}$ coordinates, respectively; t_i and y_i are the components of the traction vector and position vector on the surface of the ellipsoid in $\{\mathbf{m_1},\mathbf{m_2},\mathbf{m_3}\}$ coordinates, respectively. Note that the translational degrees-of-freedom (DOF) decouple; they are coupled to vibration through the fluid.

A.2. The water walking mode

From our experimental observations, the water walking mode appears to consist of a rigid body rotation with the sphere holding steady, deformed principal stretches; we also allow for the possibility that the principal axes rotate with steady rotation rate $\dot{\alpha}$. Therefore, we look for a steady-state solution in which the sphere is undergoing a rigid body rotation with constant $\dot{\psi}$ and $\dot{\alpha}$, with all other time derivatives and second time derivatives vanishing and all tractions vanishing. In this case, equations 25-28 are satisfied trivially and equations 29-30 reduce to

$$\delta\dot{\lambda}_1 : \quad \frac{4\pi}{15}\rho_s R^5\left[-2\dot{\alpha}^2(\lambda_1-\lambda_2)+2\dot{\alpha}\dot{\psi}(\lambda_1-\lambda_2)-\lambda_1\dot{\psi}^2\right]+\frac{4\pi}{3}GR^3\left(\lambda_1-\frac{\lambda_2}{(\lambda_1\lambda_2)^3}\right)=0$$

$$\delta\dot{\lambda}_2 : \quad \frac{4\pi}{15}\rho_s R^5\left[2\dot{\alpha}^2(\lambda_1-\lambda_2)-2\dot{\alpha}\dot{\psi}(\lambda_1-\lambda_2)-\lambda_2\dot{\psi}^2\right]+\frac{4\pi}{3}GR^3\left(\lambda_2-\frac{\lambda_1}{(\lambda_1\lambda_2)^3}\right)=0$$

Adding these equations results in

$$\frac{4\pi}{15}\rho_s R^5\left[-\dot{\psi}^2(\lambda_1+\lambda_2)\right]+\frac{4\pi}{3}GR^3\left[\lambda_1+\lambda_2-\frac{\lambda_1}{(\lambda_1\lambda_2)^3}-\frac{\lambda_2}{(\lambda_1\lambda_2)^3}\right]=0 \tag{31}$$

which reduces to

$$\dot{\psi}=\sqrt{\frac{5G}{\rho_s R^2}\left(1-\frac{1}{(\lambda_1\lambda_2)^3}\right)} \tag{32}$$

which requires $(\lambda_1\lambda_2)^3 \geq 1$ or $\lambda_1 \geq 1/\lambda_2$ for real solutions. Equation 32 thus describes a sphere in steady-state translation and rotation that holds a constant deformed shape described by $\lambda_1\lambda_2$. Equation 32 can be rearranged to

$$\lambda_1\lambda_2=\left(\frac{1}{1-\left(\dot{\psi}/\sqrt{\frac{5G}{\rho_s R^2}}\right)^2}\right)^{\frac{1}{3}} \tag{33}$$

A.3. Tracking the lowest point on the sphere with deformation model

It was shown in Belden et al. 2016 that the governing equations 25-30 permit a different steady state mode than that described above in which $\dot{\psi}=0$, but in which the principal axes $\{\mathbf{m_1},\mathbf{m_2},\mathbf{m_3}\}$ rotate with constant angular rate $\dot{\alpha}=\pm\sqrt{\frac{5G}{2\rho_s R^2}}$, which is proportional to the term in the denominator of equation 33. They also showed experimentally that $\dot{\alpha}\propto\sqrt{\frac{G}{\rho_s R^2}}$ for single impact events. Furthermore, we note that in the steady-state rotation case derived in section *A.2*, with constant $\lambda_1\lambda_2$, equations 29-30 actually permit a steady state value of $\dot{\alpha}$, in the case that all other derivatives and tractions vanish. Thus, the term $\dot{\psi}/\sqrt{\frac{5G}{\rho_s R^2}}$ in equation 33 can be thought of as the ratio of the steady state rigid body rotation rate to the steady state angular rotation rate of the principal axes.

It seems likely that what is actually observed in the field experiments is the sphere in a state with both nearly steady state and non-zero $\dot{\psi}$ and $\dot{\alpha}$. We also observe that the sphere tends to be oblong with $\lambda_1>\lambda_2$, but with each nearly steady. To analyze the sphere in this state, we consider the deformation for different values of $\dot{\psi}/\sqrt{\frac{5G}{\rho_s R^2}}$. Consider tracking a single point on the sphere perimeter $\mathbf{x}$, and that the sphere experiences a rigid rotation $\mathbf{Q}$ and a volume preserving stretch $\mathbf{V}$ (see Fig. 1). The final position of the point $\mathbf{x}$, in the $\{\mathbf{e_1},\mathbf{e_2},\mathbf{e_3}\}$ coordinate frame, is defined by

$$\mathbf{y}=\mathbf{d}+\mathbf{PVQx} \tag{34}$$

For a point in the $\mathbf{e_1}-\mathbf{e_2}$ plane, $\mathbf{x}$ is defined as

$$\mathbf{x}=R\cos\gamma\,\mathbf{e_1}+R\sin\gamma\,\mathbf{e_2}. \tag{35}$$

Following rotation and deformation, the position of this point relative to the COM is

$$\mathbf{y}-\mathbf{d}=\begin{bmatrix}\lambda_1\cos\alpha & -\lambda_2\sin\alpha & 0\\ \lambda_1\sin\alpha & \lambda_2\cos\alpha & 0\\ 0 & 0 & 1\end{bmatrix}\begin{bmatrix}\cos\psi & -\sin\psi & 0\\ \sin\psi & \cos\psi & 0\\ 0 & 0 & 1\end{bmatrix}\begin{bmatrix}R\cos\gamma\\ R\sin\gamma\\ 0\end{bmatrix}. \tag{36}$$

Letting $\sin() = s_{()}$ and $\cos() = c_{()}$, equation 36 becomes

$$\mathbf{y}-\mathbf{d}=\begin{bmatrix}R(\lambda_1 c_\alpha c_\psi c_\gamma-\lambda_1 c_\alpha s_\psi s_\gamma-\lambda_2 s_\alpha s_\psi c_\gamma+\lambda_2 s_\alpha c_\psi s_\gamma)\\ R(\lambda_1 s_\alpha c_\psi c_\gamma-\lambda_1 s_\alpha s_\psi s_\gamma-\lambda_2 c_\alpha s_\psi c_\gamma+\lambda_2 c_\alpha c_\psi s_\gamma)\\ 0\end{bmatrix}. \tag{37}$$

To find the lowest point on the sphere, we seek the of γ that minimizes the $\mathbf{e_2}$ component of $\mathbf{y}-\mathbf{d}$. Thus we find a solution for the expression

$$\frac{\partial}{\partial\gamma}\left[s_\gamma R\left(\lambda_2 c_\alpha c_\psi-\lambda_1 s_\alpha s_\psi\right)+c_\gamma R\left(\lambda_2 c_\alpha s_\psi+\lambda_1 s_\alpha c_\psi\right)\right]=0. \tag{38}$$

The derivative of equation 38 is

$$c_\gamma R\left(\lambda_2 c_\alpha c_\psi-\lambda_1 s_\alpha s_\psi\right)-s_\gamma R\left(\lambda_2 c_\alpha s_\psi+\lambda_1 s_\alpha c_\psi\right)=0. \tag{39}$$

We then solve for γ to get

$$\gamma=\tan^{-1}\left\{\frac{\lambda_2 c_\alpha c_\psi-\lambda_1 s_\alpha s_\psi}{\lambda_2 c_\alpha s_\psi+\lambda_1 s_\alpha c_\psi}\right\}. \tag{40}$$

Appendix B. Maximum skipping distance

Finally, we look for any correlation between number of skips N and distance traveled d in figure 2(a). There seems to be little correlation, which is supported by qualitative observations. For example, the largest value for distance recorded was $d = 164$ m; though this sphere was difficult to see when it landed so far away, and may have skipped an extra time prior to entry, the ball clearly skipped less than 10 times, which is at the low end of N. On the other hand, the event with the greatest number of skips $N = 124$, traveled just under 90 m before entering the water. Even when only considering the traditional skipping events in the figure 2(b), there is not an observable trend between N and d. However, one clear conclusion from figure 2(a) is that when striving to maximize number of skips N, one should strive for a water walking type 2 skipping event.

References

1. Bower, A. F. *Applied mechanics of solids* (CRC press, 2009).

2. *Guinness Book of World Records* (Stamford, CT: Guinness Media, 2018).

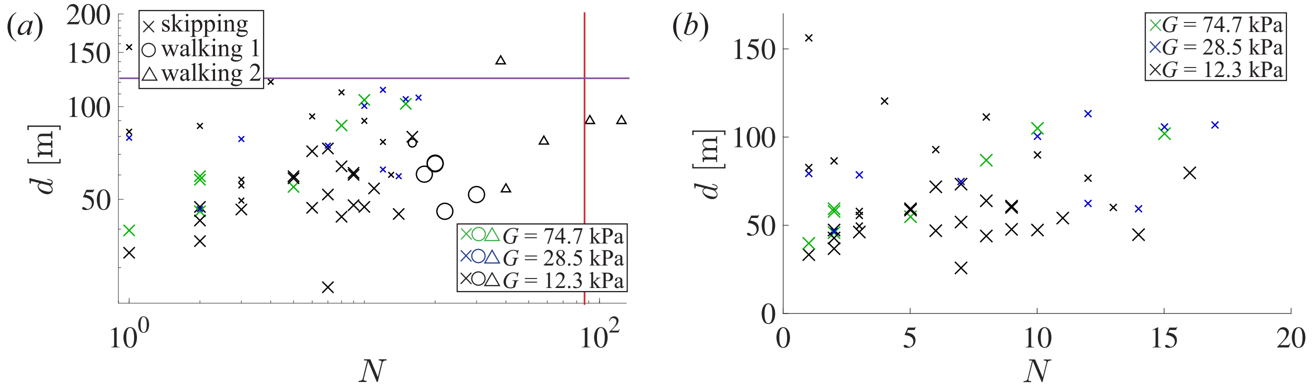

Figure 2. (*a*) Distance to water entry d is presented as a function of number of skips N for all measured multi-skip events. Water walking events exhibit significantly higher values for N. The vertical red line marks the current world record for stone skipping of 88 skips and the purple line marks the world record for skipping distance of 121.8 m[2]. (*b*) Distance to water entry d is plotted against N for only traditional skipping events.
